# Bibliography

**Published:** 1900-09-15

**Authors:** 


### BIBLIOGRAPHY.

*Facts, Fads and Fancies About the Teeth*, is a collection of literary "odds and ends" on the subject of dentistry. It is compiled by Dr. Henry L. Ambler, of Cleveland, Ohio, and is printed in a book of about three hundred octavo pages by the Helman-Taylor Company, of Cleveland, Ohio. The introduction is in the form of a poem of four cantos, "and it's all on account of the teeth." It was written by the editor of the *Ohio Dental Journal.* The poem is good enough to capture the prize offered by the editor of the *Indiana Dental Journal*, except that it fails to conform to the specifications. The book contains much of the usual chaff and coarse wit of the newspaper scribbler, and many things which might just as well have been left in obscurity, so far as any good purpose they will ever serve. In fact, there are many things to which we seriously object, because they lack refined humor and dignity of purpose and tend to discredit the profession.

The author's vanity may be stimulated by printing in the first chapter his private correspondence, in which he is called the "King of Dentists" by a patient seeking an appointment to be "punished." Such comments by patients may be courteously endured in the privacy of business relations, but are almost intolerable when publicly proclaimed, even as wit. The author expresses the desire that this work may entertain some one during a weary or leisure moment, and there are many things in the book well worth reading. There is much that is witty, also much that is entertaining, and some things instructive. It is not a book for the public reading-room, or even for the center table of the dentist's reception room; but any dentist with a discriminating taste will enjoy reading it and will value it for the many interesting records nowhere else to be found. The chapters on Proverbs and Quotations from the Bible and Shakespeare are of interest, as are also the prose and poetical quotations and the chapter on tooth lore. The book is well printed and attractively bound; the illustrations are well done, but not such as we should select for such a work. Dr. Ambler is to be commended for his laborious task in collecting the material for such a book. We trust that future editions may more accurately sense our professional characteristics and idiosyncrasies. The book would be more enjoyable and have a longer existence even if it might not be quite so funny.

